# All-cause mortality among people with serious mental illness (SMI), substance use disorders, and depressive disorders in southeast London: a cohort study

**DOI:** 10.1186/1471-244X-10-77

**Published:** 2010-09-30

**Authors:** Chin-Kuo Chang, Richard D Hayes, Matthew Broadbent, Andrea C Fernandes, William Lee, Matthew Hotopf, Robert Stewart

**Affiliations:** 1 King's College London, Section of Epidemiology, Dept of Health Service and Population Research, Institute of Psychiatry, London, UK; 2 King's College London, Academic Dept Psychological Medicine, Institute of Psychiatry, London, UK

## Abstract

**Background:**

Higher mortality has been found for people with serious mental illness (SMI, including schizophrenia, schizoaffective disorders, and bipolar affective disorder) at all age groups. Our aim was to characterize vulnerable groups for excess mortality among people with SMI, substance use disorders, depressive episode, and recurrent depressive disorder.

**Methods:**

A case register was developed at the South London and Maudsley National Health Services Foundation Trust (NHS SLAM), accessing full electronic clinical records on over 150,000 mental health service users as a well-defined cohort since 2006. The Case Register Interactive Search (CRIS) system enabled searching and retrieval of anonymised information since 2008. Deaths were identified by regular national tracing returns after 2006. Standardized mortality ratios (SMRs) were calculated for the period 2007 to 2009 using SLAM records for this period and the expected number of deaths from age-specific mortality statistics for the England and Wales population in 2008. Data were stratified by gender, ethnicity, and specific mental disorders.

**Results:**

A total of 31,719 cases, aged 15 years old or more, active between 2007-2009 and with mental disorders of interest prior to 2009 were detected in the SLAM case register. SMRs were 2.15 (95% CI: 1.95-2.36) for all SMI with genders combined, 1.89 (1.64-2.17) for women and 2.47 (2.17-2.80) for men. In addition, highest mortality risk was found for substance use disorders (SMR = 4.17; 95% CI: 3.75-4.64). Age- and gender-standardised mortality ratios by ethnic group revealed huge fluctuations, and SMRs for all disorders diminished in strength with age. The main limitation was the setting of secondary mental health care provider in SLAM.

**Conclusions:**

Substantially higher mortality persists in people with serious mental illness, substance use disorders and depressive disorders. Furthermore, mortality risk differs substantially with age, diagnosis, gender and ethnicity. Further research into specific risk groups is required.

## Background

The potential impact of mental disorders on mortality has been increasingly recognised in recent years. People with serious mental illnesses (SMI, including schizophrenia, schizoaffective disorder, bipolar disorder, and depressive psychosis) live with health disparities, not only resulting from social dysfunction, stigma, and direct consequences of psychopathology, but also potentially from the deleterious physical consequences of long-term antipsychotic use and adverse lifestyle choices (e.g. smoking, diet, illicit drug use, and physical inactivity), particularly contributing to cardiovascular mortality [1-5].

Higher mortality has been found for people with serious mental illness (SMI) in all age groups. In the US, life expectancy for people with SMI from public mental health agencies in eight states for 1997 through 2000 was reported to be at least 30% shorter than that for the general population [6]. In the UK, any psychiatric diagnosis was associated with a 65% higher than expected total mortality in one case register study [7], and a three-fold elevated mortality was found for coronary heart disease in young adults with SMI in a primary care setting, as well as an almost two-fold elevation for those aged 50-75 years, and a more than two-fold increased stroke mortality in all age groups [8]. Furthermore, a greater than 10-fold increased risk of suicide mortality was found among the same groups, itself associated with increased consultation rates, antidepressant prescriptions, and residence in less deprived areas [9].

Despite recent improvements in health care systems, there has been little evidence for benefits on prolonging life expectancy in people with SMI [5,10,11]. Instead, a recent systematic review suggested a widening mortality gap over recent decades with the pattern of change suggesting a failure to benefit from population improvements in health rather than an actual increased case fatality rate [11]. Given this persistence of excess mortality, particularly in younger age groups, there is an imperative need for further investigations in this area [10,12,13]. Preliminary strategies for preventing premature deaths among people with SMI have been proposed, including the management of suicide risk and physical illness, minimizing polypharmacy, and improving accessibility to physical health care [1]. However, there is limited evidence upon which to base these. Previous analyses of the UK General Practice Research Database (GPRD) have only reported associations between any SMI and mortality and did not differentiate between disorders [8]. Further evidence is required to clarify the characteristics of people with SMI who are at highest risk of mortality.

We therefore investigated excess mortality for people with individual disorders within the SMI grouping, as well as for depressive and substance use disorder diagnoses, drawing on data from the South London and Maudsley NHS Foundation Trust Biomedical Research Centre (SLAM BRC) Case Register, which covers comprehensive secondary mental healthcare provision to a large geographically defined community.

## Methods

### Setting and study population

The SLAM BRC Case Register provides anonymised in-depth information derived from electronic medical records relating to secondary mental health care, which includes all specialist care (i.e. apart from that provided by general practitioners) for hospitalization, outpatient care, a broad profile of community care models (both acute care and rehabilitation), psychiatric liaison services to general hospitals, and forensic mental health services. The protocol for this case register has been described in detail in an open-access publication [14]. SLAM provides comprehensive secondary mental health care to a population of approximately 1.3 million residents of four London boroughs (Lambeth, Southwark, Lewisham and Croydon) as well as tertiary care national referral units. Under the British National Health Service (NHS), all secondary mental healthcare within these four boroughs is provided at no cost to consumers by SLAM, the only exception being people seeking exclusively private healthcare [15]. Electronic clinical records have been used comprehensively across all SLAM services since 2006 and the Case Register Interactive Search (CRIS) system was developed in 2008 to allow searching and retrieval of anonymised information with over 150,000 cases currently represented on the system. CRIS was approved as a dataset for secondary analysis by Oxfordshire Research Ethics Committee C, reference 08/H0606/71.

This analysis focused on recorded mortality over a three-year period from 2007 to 2009. Cases were included if they had had contact with SLAM services (a referral, discharge, or case note entry) from 1^st ^Jan, 2007 to 31^st ^Dec, 2009 and had received an SMI, substance use disorder, depressive episode, or recurrent depressive disorders diagnosis before or during that time. In the duration, routine mortality monitoring was carried out on these patient records, hence patients were "at risk" of death during this period of time. Date of birth and gender were routinely recorded and verified on the SLAM electronic patient electric records in designated fields. Age was calculated at the 1^st ^of July, 2008 (i.e. the mid-point of the "at risk" period). All those who were under the age of 15 at this date were excluded from the analyses.

### Mortality and covariates

NHS number is a unique identifier for UK NHS records, all death certifications are linked to this identifier at a national level, and primary and secondary health service providers are required by law to keep records up to date with respect to this. A list of deceased patients associated with SLAM is downloaded on a monthly basis for the "Service User Death Report" from "the Spine" provided by NHS Care Records Service for whom has been marked as deceased by an update from another patient demographic system. For non-active previous service users, this was taken to be the date of death. Further checking routinely occurs for active service users to corroborate the death. The last monthly download was 31 March, 2010 and completed on 7 April, 2010. Diagnoses recorded in the SLAM BRC Case Register were based on the 10^th ^edition of the World Health Organization International Classification of Diseases (ICD-10). In this analysis, patients were classified as having an SMI if they had received at least one of the following diagnoses (identified by corresponding ICD-10 codes) during their time in contact with SLAM services: schizophrenia (F20), schizoaffective disorders (F25), and bipolar affective disorder (F31). Substance use disorders (F10 to F19), depressive episode (F32) and recurrent depressive disorder (F33) were also used for analyses. Separate analyses were carried out for each disorder or grouping - i.e. those with more than one primary diagnosis during the follow-up period could appear in more than one 'case' group. Ethnic group classifications applied in the Case Register were: "White British", "Other white background", "East Asian", "South Asian", "African and other black background", "Caribbean", and "Mixed, unknown, and others".

### Statistical analysis

Standardized mortality ratios (SMRs) were calculated by Stata for the three-year observation period, using the number of deaths observed in SLAM records in these three years as the numerator. The denominator was the expected number of deaths in a year estimated by age- and/or gender-specific mortality statistics for the England and Wales population in 2008 multiplied by three [16]. SMRs were calculated using age strata (namely, 15-19, 20-24, 25-29, 30-34, 35-39, 40-44, 45-49, 50-54, 55-59, 60-64, 65-69, 70-74, 75-79, 80-84, 85-89, and 90+) and also by gender and ethnicity strata for specific mental disorders. Because the geographic catchment area providing the majority of SLAM referrals was restricted to southeast London, additional sensitivity analyses were carried out using mortality statistics for London alone in 2008, as published by the Office of National Statistics [16], although, because of restrictions in these source data, wider age strata had to be applied for standardization (15-24, 25-34, 35-44, 45-54, 55-64, 65-74, 75-84, and 85+).

## Results

A total of 38,066 cases were identified using CRIS with a primary diagnosis, recorded before the end of March 2010, of substance use disorder, schizophrenia, schizoaffective disorder, bipolar affective disorder, depressive episode, or recurrent depressive disorder. Among these, 5,902 cases became inactive to SLAM services before 1 January, 2007 and remained inactive over the follow-up period and were thus excluded from further analyses. Those younger than 15 years old at the mid-point of 2008 or missing date of birth were also excluded (n = 445). Therefore, a total of 31,719 cases of interest with 1,370 deaths were identified. Of the sample, 1,680 (5.3%) had two diagnoses of interest before the end of 2009, 121 (0.4%) had three diagnoses and six (0.02%) had four diagnoses. The most common combinations of diagnoses applied on different occasions in the same individual were schizophrenia and schizoaffective disorders (n = 421), followed by substance use disorders and depressive episode or recurrent depressive disorder (n = 352).

**Figure 1:**
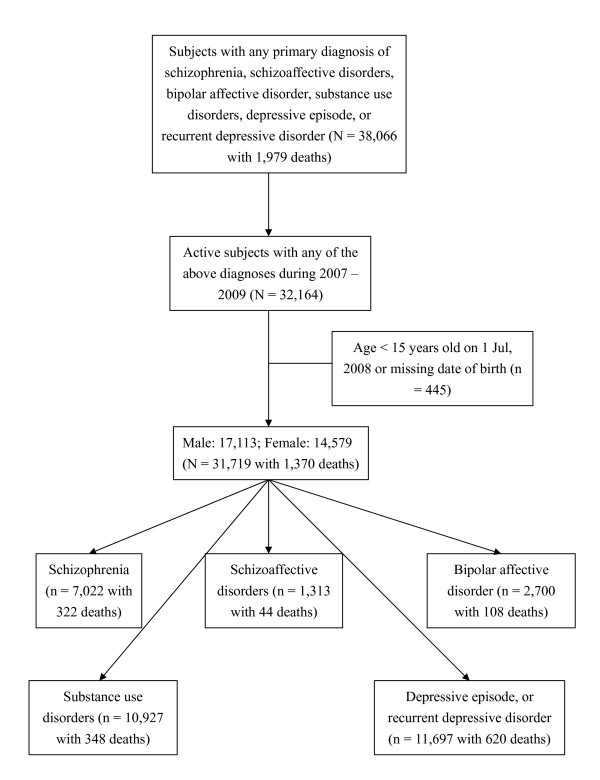
**Sample selection and diagnosis cohorts investigated**.

Figure 1 summarizes the data retrieval process and number of deaths in each group of interest. Age-standardised mortality ratios (SMRs) of mental disorder diagnoses for the total cohort and stratified by gender are displayed in Table 1. Mortality was significantly elevated for all disorder groups - highest overall for substance use disorders, followed by schizoaffective disorder, schizophrenia, bipolar affective disorder and depressive episode. The ranking of these disorder-specific SMRs was similar between men and women, except that, in women, there were stronger associations with schizoaffective and bipolar affective disorders compared to schizophrenia. The SMR for any SMI was stronger in men than women, principally because of the marked gender difference for schizophrenia. SMRs for depressive disorders were also higher for men compared to women.

**Table 1:** Age-standardised mortality ratios (SMRs), stratified by gender, for mental disorder diagnoses in SLAM^#^

	Standardised mortality ratio for deaths in 2007-09 (95% CI, number of deaths)		
	
Diagnosis	Total	Male^	Female^
**SMI - any**	2.15 (1.95-2.36, n = 446)*	2.47 (2.17-2.80, n = 243)*	1.89 (1.64-2.17, n = 203)*
Schizophrenia (F20)	2.25 (2.01-2.51, n = 322)*	2.78 (2.40-3.19, n = 196)*	1.74 (1.45-2.07, n = 126)*
Schizoaffective disorders (F25)	2.52 (1.83-3.39, n = 44)*	2.35 (1.35-3.86, n = 16)*	2.88 (1.91-4.16, n = 28)*
Bipolar affective disorder (F31)	1.95 (1.60-2.35, n = 108)*	1.76 (1.27-2.37, n = 43)*	2.21 (1.71-2.82, n = 65)*
**Substance use disorders (F10 - F19)**	4.17 (3.75-4.64, n = 348)*	3.60 (3.17-4.07, n = 254)*	4.67 (3.78-5.72, n = 94)*
**Depressive episode and recurrent depressive disorder (F32 - F33)**	1.29 (1.19-1.40, n = 620)*	1.53 (1.36-1.72, n = 284)*	1.18 (1.06-1.31, n = 336)*

# Compared to the population of England and Wales in 2008

* P-value <0.05

^ Standardised by gender- and age-specific mortality rates, separately (27 missing values for gender)

Table 2 displays age- and gender-standardised mortality ratios stratified by ethnic group. SMRs for SMI diagnoses were comparable in size across all groups apart from non-significant findings in the smallest East and South Asian groups. SMRs for substance use disorders appeared more heterogeneous, although confidence intervals were wide and overlapping.

**Table 2:** Age- and gender-standardised mortality ratios (SMRs), stratified by ethnicity, for mental disorder diagnoses in SLAM^#^

	Standardised mortality ratio for deaths in 2007-09 (95% CI, number of deaths)		
	
Ethnic group	SMI (F20, 25, & 31)	Substance use disorders (F10 - F19)	Depressive episode and recurrent depressive disorder (F32 - F33)
White British	1.97 (1.72-2.24, n = 224)*	3.94 (3.47-4.46, n = 250)*	1.30 (1.18-1.43, n = 455)*
Other white back ground	2.28 (1.74-2.92, n = 61)*	4.18 (3.08-5.54, n = 48)*	1.29 (0.99-1.64, n = 63)
East Asian	1.63 (0.65-3.35, n = 7)	0.87 (0.02-4.82, n = 1)	0.77 (0.25-1.81, n = 5)
South Asian	1.64 (0.79-3.02, n = 10)	6.55 (2.83-12.91, n = 8)*	1.93 (1.05-3.23, n = 14)*
African and other black background	3.51 (2.61-4.62, n = 51)*	2.23 (1.02-4.23, n = 9)*	1.07 (0.46-2.11, n = 8)
Caribbean	2.06 (1.58-2.63, n = 64)*	2.73 (1.18-5.38, n = 8)*	1.52 (1.04-2.15, n = 32)*
Mixed, unknown, and others	3.21 (2.15-4.62, n = 29)*	3.76 (2.41-5.60, n = 24)*	1.39 (1.01-1.88, n = 43)*

# Compared to the population of England and Wales in 2008

* P-value < 0.05

As displayed in Table 3, SMRs for all disorders diminished in strength with increasing age. Even so, all disorders remained significant predictors of mortality in the oldest (65+ years) age stratum.

**Table 3:** Age-standardised mortality ratios (SMRs), stratified by age group, for mental disorder diagnoses in SLAM^#^

	Standardised mortality ratio for deaths in 2007-09 (95% CI, number of deaths)		
	
Diagnosis	15 - 44 years old	45 - 64 years old	65+ years old
**SMI - any**	4.47 (3.49-5.64, n = 71)*	3.10 (2.61-3.66, n = 140)*	1.60 (1.40-1.82, n = 235)*
Schizophrenia (F20)	4.73 (3.52-6.22, n = 51)*	3.44 (2.82-4.16, n = 106)*	1.63 (1.39-1.89, n = 165)*
Schizoaffective disorders (F25)	3.96 (1.81-7.52, n = 9)*	2.71 (1.48-4.55, n = 14)*	2.10 (1.30-3.21, n = 21)*
Bipolar affective disorder (F31)	4.09 (2.38-6.54, n = 17)*	2.58 (1.77-3.64, n = 32)*	1.51 (1.15-1.95, n = 59)*
**Substance use disorders (F10 - F19)**	6.81 (5.77-7.98, n = 153)*	4.40 (3.70-5.20, n = 139)*	1.91 (1.44-2.48, n = 56)*
**Depressive episode and recurrent depressive disorder (F32 - F33)**	3.21 (2.40-4.20, n = 53)*	1.75 (1.35-2.22, n = 66)*	1.18 (1.08-1.28, n = 501)*

# Compared to the population of England and Wales in 2008

* P-value < 0.05

Secondary sensitivity analyses were carried out by standardising with London mortality in 2008 (details not shown). In summary, SMRs were comparable and, if anything, slightly stronger than those displayed in Tables 1, 2 and 3. For example, the re-calculated SMRs for any SMI were 2.21 for the total sample, 2.49 for men and 1.98 for women. The respective SMRs were 4.28, 3.70 and 4.76 for substance use disorders and 1.42, 1.72 and 1.29 for depressive disorders.

## Discussion

In this analysis, people with mental disorder diagnoses who had had contact with secondary mental healthcare services, had substantially higher mortality than expected in all diagnostic groups examined. The calculated SMRs varied modestly by gender and substantially by age, and also fluctuated markedly across different ethnic groups, which might be caused by small size in specific populations.

People with SMI have substantially higher than expected mortality in all age groups. The raised risk of mortality in these cohorts is consistent with previous studies, indicating that mortality rates among individuals with SMI are higher than that of the general community [6-8]. A previous longitudinal register-based study with the maximum follow up of 18 years in the UK investigated 'any psychiatric diagnosis' as an exposure and found this to be associated with a 65% higher than expected total mortality [7]. In our analysis, the SMRs of those with SMI suggested a more than two-fold higher mortality, although this may reflect a focus on the more severe mental disorders represented within the SMI label. Causal pathways between mental disorder and mortality have yet to be fully elucidated and are likely to be multiple. While suicide and accidents/violence are important considerations for clinical services, research has tended to show mortality increases across all major causes, including cardiovascular diseases (heart attack and stroke) [11]. These may be influenced by direct effects of mental disorder symptoms (for example on suicide and accidents). Pathways may also reflect adverse lifestyle factors influenced by the presence of mental disorders and themselves responsible for associations with mortality; these include worse nutrition, physical inactivity, alcohol use, smoking, and illicit drug use [2-4]. Adverse effects from psychotropic (particularly antipsychotic) medication have also received increasing consideration in terms of their role in raised mortality as an outcome [17]. Specifically, antipsychotic agents are often prescribed long term and may increase the risks of diabetes mellitus and cardiovascular diseases with events including QT-interval prolongation, ventricular arrhythmias, pulmonary embolus, atherosclerosis, and sudden cardiac death [4,18-20]. The analyses presented here were not intended to elucidate causal pathways, but rather to constitute the first stage in a series of investigations of these issues with future studies clarifying further the role of socioeconomic status, education and cognitive abilities which are all associated with higher mortality and might confound the reported associations [21,22].

Higher mortality in people with schizophrenia has been recognised since the 1930 s [11]. Nonetheless, as stated earlier, this mortality gap has remained stubbornly unchanged [10], and may even have increased over time [5,11], despite developments in mental health services and the introduction of better tolerated antipsychotic agents. Previous studies suggest that around two-thirds of the excess mortality among people with schizophrenia may be attributable to natural causes, as discussed above [23], with the remaining one third being due to suicide and other unnatural causes [23-26]. Regarding bipolar disorder, a non-significant raised SMR for all causes of death was reported in another study performed in South London with a more limited sample size (239 cases with 42 deaths over 19 years) [27]. Regarding SMRs for cases with depressive episode or recurrent depressive disorder, a particularly high mortality risk was identified among the younger age stratum (15-44 year olds, Table 3). However, the SMRs for depression were relatively low [28]. This finding may be due to the fact that people with depression known to secondary care are not representative of those with the disorder in the community and, in particular, that referral bias for secondary care favours those with relatively good health or higher social class [29,30].

Our findings are, we believe, novel in the presentation of SMRs stratified by age, gender, and ethnicity. Effect modification, where demonstrated, may provide at least some supportive evidence regarding causal pathways since it indicates uneven distribution of risk; however, such conclusions can only be drawn tentatively and require confirmatory investigation. Gender differences in the associations of interest might reflect different levels of environmental support (for example, lower social support for men with schizophrenia might account for the higher SMR in that group), or might reflect gender differences in the severity of the condition in question or in the level of comorbidity with other physical, mental or personality disorder [31]. The substantial differences in SMRs between ethnic groups again suggest that some of the associations between SMI and mortality may be socially mediated, although confidence intervals were wide for many groups and negative findings should be viewed with caution. The diminution of mortality risk with age may reflect survival effects, with those surviving with serious mental disorder to age 65+ being relatively healthier in other respects. Alternatively, people with SMI in older age ranges may be more likely to remain in contact with mental health services and adhere to treatment, whether for mental or physical disorders. However, the age diminution could also reflect differences in the nature of the underlying disorders, such as symptomatic differences between early- and late-onset schizophrenia, or differences in substances misused in younger and older adults. Further, it may reflect the excess risk of SMI being additive rather than multiplicative, so it is obscured by deaths from other causes in older age groups [7,20].

This investigation has a number of strengths. We were able to draw on a large numbers of case records from the largest single provider of secondary mental healthcare in Europe. The National Health Service presents additional contextual advantages because of its near-total coverage of all aspects of healthcare in the UK. This investigation was able to draw on complete electronic clinical records of more than 31,000 patients diagnosed with SMI, depression or substance use disorders providing the statistical power to be able to differentiate between disorders as well as explore subgroup-specific mortality risk.

However, potential limitations should also be considered. First of all, confounders other than age and gender might still exist. Case registers derived from secondary healthcare offer particular advantages for investigating "high penetrance" disorders - i.e. those like schizophrenia and bipolar affective disorder where the chances of secondary care contact are high. For lower penetrance disorders, inferences need to be more cautious and both depressive and substance use disorders fall into this category - i.e. cases known to secondary care may reflect more severe primary disorders, comorbidity, environmental disadvantage or referral biases. Prevalence bias is an additional issue which needs consideration, in that the cases known to a service within a given time period are likely to be dominated by those with long and relapsing clinical courses - they therefore cannot be taken to generalise to incident cases. A further methodological challenge was that service data were principally (but not entirely) derived from a single catchment area whereas expected deaths were derived from national data. However, a sensitivity analysis using London-specific data, described above, did not reveal any meaningful differences. Finally, we have not included data on causes of death and further research is required for this, as well as for investigating specific risk factors for mortality within particular disorders. Diagnostic categories overlapped since a proportion of individuals suffer from more than one mental disorder, but no attempt in this analysis was made to consider comorbidity.

## Conclusions

People with serious mental illness, substance use disorders and depressive disorders continue to be at higher risk of mortality compared to the general population. Furthermore, mortality risk differs substantially with age, diagnosis, gender and ethnicity. Further research into specific risk groups is required.

## Competing interests

The authors declare that they have no competing interests.

## Authors' contributions

All the authors listed contributed themselves in the process of hypothesis generation, data collection, statistical analyses, or manuscript preparation, and fulfilled the criteria for authorship. CKC and RH carried out the data retrieval, statistical analyses, and manuscript drafting. AF, MB, WL and RS participated in the hypothesis generation, data management, and assistant on manuscript preparation. CKC, RH, MH and RS conceived of the study, participated in its design, and implemented the project. All the authors read and approved the final manuscript.

## Pre-publication history

The pre-publication history for this paper can be accessed here:

http://www.biomedcentral.com/1471-244X/10/77/prepub
